# Pretreatment and Membrane Hydrophilic Modification to Reduce Membrane Fouling

**DOI:** 10.3390/membranes3030226

**Published:** 2013-09-04

**Authors:** Wen Sun, Junxia Liu, Huaqiang Chu, Bingzhi Dong

**Affiliations:** State Key Laboratory of Pollution Control and Resource Reuse, School of Environmental Science and Engineering, Tongji University, Shanghai 200092, China; E-Mails: sunwen-sophia@163.com (W.S.); whjunxia@163.com (J.L.); chq123zl@hotmail.com (H.C.)

**Keywords:** pretreatment, NOM, membrane hydrophilic modification, membrane fouling, drinking water

## Abstract

The application of low pressure membranes (microfiltration/ultrafiltration) has undergone accelerated development for drinking water production. However, the major obstacle encountered in its popularization is membrane fouling caused by natural organic matter (NOM). This paper firstly summarizes the two factors causing the organic membrane fouling, including molecular weight (MW) and hydrophilicity/hydrophobicity of NOM, and then presents a brief introduction of the methods which can prevent membrane fouling such as pretreatment of the feed water (e.g., coagulation, adsorption, and pre-oxidation) and membrane hydrophilic modification (e.g., plasma modification, irradiation grafting modification, surface coating modification, blend modification, *etc.*). Perspectives of further research are also discussed.

## 1. Introduction

The membrane separation technology with different pore size membrane filter for water and water pollutants removal began to develop in the 1970s and developed rapidly from the 1990s. For the popularization and application process of membrane, membrane fouling has been the “bottleneck”. The membrane fouling causes the permeation flux decline and increases running power cost and the replacement cost of the membrane, which all increased the cost of water produce. Therefore, the effective control of membrane fouling has been the forefront and hot issues in the field of water treatment [1]. It is generally believed that the main reasons for the formation of membrane fouling include pore clogging, contaminants adsorption, the formation of the gel layer, concentration polarization, and cake layer formation [2,3]. The membrane fouling can also be divided into organic fouling, inorganic fouling, and microbial contamination based on the fouling substances [4]. The natural organic matter (NOM) is a complex organic substances in natural water, including colloidal polysaccharide, humic acid, fatty acids, proteins, *etc.* [5,6,7], which are the main substances for membrane fouling in surface water treatment [5]. Membrane fouling by NOM can not be restored only through physical scrub like water and air wash, and the complex chemical cleaning process or the pretreatment process are needed to maintain the long-term stable operation of a membrane. This paper summarized the two factors causing the organic membrane fouling, including molecular weight (MW) and hydrophilicity/hydrophobicity of NOM, and then a brief introduction of the methods to prevent membrane fouling were present, as well as the perspectives of further research.

## 2. Factors Causing Membrane Fouling

### 2.1. Hydrophilicity/Hydrophobicity of NOM

Many scholars studied the different components of organics, and found that the hydrophilicity/hydrophobicity of organic matters played a key role in membrane fouling and membrane flux decline. Lim *et al.* summarized organic compounds assigned to a particular fraction according to their chain length and functional groups [8] (Table 1, adapted from Buchanan *et al.* [9]). Carroll *et al.* found that the netural hydrophilic organics from surface water mainly induced the fouling of microfiltration [10]. Fan *et al.* researched three different surface waters in Australia, and found that the order of four components for micro-membrane fouling was: netural hydrophilic fraction > strong hydrophobic fraction > weak hydrophobic fraction > polar hydrophilic fraction [11]. Gray *et al.* found that the neutral hydrophilic organics and polar hydrophilic organics in a lake in Australia could form a gel layer on the surface of microfiltration membrane and induced rapid flux decline, while the hydrophobic component could only caused a slow flux decline. However, completely different conclusions were also reported [12]. Chen *et al.* found the hydrophilic organics from river water only caused a slow flux decline of ultrafiltration membrane, while the hydrophobic organics of macromolecules caused sharp flux decline [13]. The membrane surface parameters, hydrophobicity, charge, morphology, and roughness can be critical to the mechanism of fouling which, in turn, will affect product quality and performance [14,15]. In addition, it is indicated that the charge can be influenced significantly by the choice of cleaning agent and membrane. Results of both zeta-potential and flux data suggested that for very rough membranes the influence of charge becomes negligibly small, thereby not playing any role in influencing subsequent fouling [16]. 

**Table 1:** Proposed composition of humic acid fractions separated using rapid fractionation technique (adapted from Buchanan *et al.* [9]).

Fraction	Organic compounds
Hydrophobic (VHA and SHA)	
Acid	Soil fulvic acids, C_5_–C_9_ aliphatic carboxylic acids,1- and 2-ring aromatic carboxylic acids, 1- and 2-ring phenols
Base	1- and 2-ring aromatics (except pyridine), proteinaceoussubstances
Neutral	Mixture of hydrocarbons, >C_5_ aliphatic alcohols, amides, aldehydes, ketones, esters, >C_9_ aliphaticcarboxylic acids and amines, >3 ring aromatic carboxylic acids and amines
Hydrophilic (CHA and NEU)	
Acid	Mixtures of hydroxy acids, C_5_ aliphatic carboxylic acids, Polyfunctional carboxylic acids
Base	Pyridine, amphoteric proteinaceous material (*i.e.*, aliphatic amino acids, amino sugars, C_9_ aliphatic amines, peptides, and proteins)
Neutral	<C_5_ aliphatic alcohols, polyfunctional alcohols, short-chain aliphatic amines, amides, aldehydes, ketones, esters; cyclic amides, polysaccharides, and carbohydrates

### 2.2. Molecular Size of Organics

The molecular size of organics has a great influence on membrane filtration performance. According to the mechanical sieving principle of membrane filtration, organic matter with relative molecular weight greater than the membrane pore size can clog the membrane pores, resulting in the decline of membrane flux; organic matter with relative molecular weight smaller than the membrane pore size will enter the inside of the membrane pores, which also affect the membrane flux. Therefore, the molecular weight of organic matters has been the focus of the study of membrane fouling. Lankes *et al.* found that organic materials with high molecular weight, such as polysaccharides and long-chain aliphatic compounds, could be retained by the membrane more easily, and aromatic compounds with medium molecular weight, such as lignin or tannic acid, could through the membrane more easily [14]. It was reported that colloidal and hydrophilic organic macromolecules (>10 kDa) from algae metabolism is the main substance that caused low-pressure membrane fouling of different hydrophilic and hydrophobic materials, which deposited on the membrane surface forming the cake layer and resulted the rapid decline of membrane flux [15,17]. Fan *et al.* also concluded that organics with macromolecules (>30 kDa) mainly caused rapidly flux decline of membrane [11]. However, some researchers believe that small organic molecules (<3 kDa) could also cause severe membrane fouling problems [18]. The impacts of organics of small molecules on the membrane flux are often subject to the influence of the chemical properties of organic matter. The effect of neutral hydrophilic organics with small molecules on membrane fouling is often greater than other small hydrophobic molecules [10]. This is because the removal of organics with small molecules membrane is not trapped by the physical sieving principle, but by the interaction force with small organic molecules [19]. Due to the pore size of the low-pressure membrane, at the micron level, the molecular sizes of dissolved organic matter are significantly smaller than the membrane pore size. Researchers suggested that the fouling of low pressure membranes resulted from the synthesis effects of macromolecules and small organic molecules, which need further investigation to get a more definitive conclusion [20]. 

### 2.3. Brief Summary

Effects of MW distribution on the reversible and irreversible fouling of immersed ultrafiltration membranes of three different materials were diffusely investigated using representative sources of natural waters. The lower MW fractions and the more hydrophilic are preferentially transmitted through the membrane pores, due to the hydrophilic components of the NOM being smaller than the hydrophobic components [21], and different MW fractions exhibit different fouling tendencies. Thus, perhaps molecular size is the most fundamental factor of membrane fouling.

## 3. Pretreatment

The low-pressure membranes have relatively large membrane pores, leading to undesirable removal efficiencies of NOM, of which the effluent cannot meet the quality of potable water. Meanwhile, pollutants in raw water can cause severe flux decline of membrane. Frequent membrane cleaning will increase operation costs as well as decrease membrane module performance and operation efficiency. Thus, a proper pre-treatment process before membrane filtration will, not only improve the treatment efficiencies of the whole system, but also alleviate membrane fouling, decrease membrane cleaning frequency, and prolong membrane life span. Several methods to prevent membrane fouling including coagulation, activated carbon adsorption, ozone oxidation, *etc.* are proposed. It is effective to reduce the accumulation of pollutants on the membrane surface and modify the interaction between membrane surface and pollutants through pretreatment measures to alleviate membrane fouling. Various pretreatment measures are summarized as follows. 

### 3.1. Coagulation Pretreatment

The coagulation-sedimentation technology is the traditional and reliable water treatment technology, which has been widely used in the water treatment field because of its low cost and ease to operate. The main utilization of this technology is to remove turbidity, but its ability to remove organic matter is limited. Commonly, organic matters with low molecular weight and large solubility organic cannot be removed effectively by the coagulation-sedimentation, which has high removal efficiencies for the hydrophobic organic matters of macromolecule in water. The pre-coagulation technologies applied before the ultrafiltration membrane can be mainly divided into two kinds, including standard coagulation and online coagulation. The online coagulation/ultrafiltration refers to the operation method that dosing coagulants before ultrafiltration without sedimentation and filtration steps after coagulation. Liang *et al.* compared three pretreatment measures including coagulation, coagulation-sedimentation, and coagulation-sedimentation-filtration combined with ultrafiltration to treat algae-abundant reservoir water, which found that the coagulation-sedimentation was the most efficient pretreatment measure [22]. Dong *et al.* [13] asserted that online coagulation could form a cake layer on the membrane surface, which was positive for the removal of natural organics so as to improve effluent quality and decrease membrane fouling. Jin *et al.* concluded based on series experiments that compared to ultrafiltration, the removal efficiencies of DOC and UV_254_ by online coagulation could improve from 28%, 40% to 53%, 78%, respectively [23]. When investigated, the controlling irreversible membrane fouling of polysulfone ultrafiltration membrane by pre-coagulation/sedimentation predicted that the pre-coagulation/sedimentation could not eliminate macromolecular organics, including polysaccharides and proteins, which were the major contributor to irreversible membrane fouling [24]. Gao *et al.* considered that the pre-coagulation put emphasis on improving filtration efficiency of ultrafiltration membrane, so it was vital to choose proper coagulants and control coagulation conditions to produce flocus that had the best coagulation-ultrafiltration treatment efficiency based on raw water qualities [4]. 

### 3.2. Adsorption Pretreatment

The combination technology of powder activated carbon (PAC)-low pressure membrane is considered as the “crystal technology” in the membrane water treatment technologies. Dosing PAC can improve removals of pollutants in the water by adsorption. Similar with coagulation pretreatment, the PAC cake layer formed on the membrane surface can also facilitate filtration efficiency. Dong *et al.* thought PAC could decrease membrane filtration resistance by changing the structure of cake layer instead of eliminating pollutants that caused membrane fouling [3]. PAC was able to decrease membrane filtration resistance and improve filtration flux to a limited degree, because PAC could only adsorb a small part of soluble organics with low molecular weight while the adsorption of macromolecular organics that affected filtration flux was poor [25]. Suzuki *et al.* investigated the composite MF system by dosing PAC with 25 μm in size to 10 g/L and PAC with 10 μm in size to 7.2 g/L respectively [26]. After dosing PAC, the decline rate of filtration flux was slowed. It was due to, compared to coagulation, PAC could adsorb macromolecular organics, which might cause membrane fouling as well as low molecular weight organics. PAC dosage for pilot test was 10 g/L and no obvious amelioration for filtration flux occurred. Sun *et al.* applied PAC column to pretreat feed of ultrafiltration and found that it could effectively adsorb soluble pollutants to alleviate membrane fouling [27]. Gai *et al.* investigated how could PAC modified microfiltration membrane quality and found that dosing PAC to 20 g/L in raw water and then constantly filtrate with a microfiltration membrane for 64 days, the transmembrane pressure was less than 20 kPa; while operating the same experiment system without dosing PAC for 48 days, the transmembrane pressure could reach to 61 kPa, of which results showed that PAC played an important role in reduction of membrane fouling [28]. In addition, Ma * et al.* [29] found that chemical irreversible membrane fouling was reduced with increasing dosage of PAC in membrane bioreactor (MBR). However, more investigations showed that PAC could not improve membrane filtration capacity and even aggravated filtration flux decline. Lin *et al.* [30] found that the pretreatment by PAC could not improve membrane filtration capacity; on the contrary, it could cause more severe membrane fouling, through investigating the effect of humus on ultrafiltration. Li *et al.* drew the same conclusion that PAC could majorly adsorb non-pollutants for the membrane, so the remaining membrane pollutants would bring more severe fouling [18]. Da Silva * et al.* used the obtained fluxes, filtrating water artificially contaminated with *E. coli* as the influent, and found that the coupling of granular activated carbon (GAC) treatment with membrane filtration could obtain a higher initial flux with GAC pretreatment working with a clean membrane and the addition of GAC could also decrease the membrane fouling [31]. Park *et al.* [32] and Milovic *et al.* [33] employed the grampositive bacterium *Staphylococcus aureus* to investigate the bactericidal effect of the coatings and chose the microbicidal surface coatings based on immobilized hydrophobic polycations. In addition, they proved that the *S.* *aureus* organism had been suitable for demonstrating the damage of cell membranes and the killing of cells (loss of culturability) upon contact with bactericidal surfaces. 

### 3.3. Oxidation Pretreatment

The molecular weight distribution of organic matter in water has a great influence on membrane fouling. Ozone is a powerful oxidant that preferentially oxidizes electron rich moieties containing double carbon bonds and aromatic alcohols [34]. It had obvious effect on modifying molecular weight distributions of organic matter [35]. Through ozone oxidation, macromolecular organics could be oxidized into small molecules and small molecules could be oxidized into inorganic matters, which could further decrease the concentration of fouling pollutants and radically reduce membrane fouling. You *et al.* found that pollutants adhered to the membrane surface could be removed by ozone oxidation, so as to alleviate membrane fouling [36]. Oh *et al.* found that after ozone oxidation, the degree of alleviation of membrane fouling was much higher than the reduced amount of organic matter concentration [37]. Thus, they reckoned that the reduction of organic matters through ozone oxidation might not be the only, or the most vital, factor for membrane fouling alleviation. The mechanisms of degradation of organic matter for membrane fouling by ozone oxidation and improvement of membrane filtration flux are still vague, in addition, whether ozone oxidation could be applied to large-scale practice is not clear as its strong oxidation corrodes membrane module. However, it is indisputable that ozone oxidation plays a positive role in reducing membrane fouling and improving the removal of organic matter. Orta *et al.* focused on determining the effect of ozone on the removal of dissolved organic matter (DOM) from a secondary effluent, and its relation with the permeated flux behavior in an ultrafiltration membrane. The results demonstrated that ozone effectively reduced fouling of the ultrafiltration membrane and improve effluent quality [34].

### 3.4. Other Pretreatments

There are other pretreatment options such as MIEX, biological treatment, and some integrated pretreatment processes to enhance membrane performance and reduce fouling. MIEX resins could remove the ions in the raw water by ion exchange [4] and remove low molecular fractions of organic matter, which was more effective than coagulation [38]. The impact of membrane properties is closely related to membrane fouling in the presence of MIEX pretreatment and research showed that MIEX pretreatment was effective for DOC removal, but less effective in controlling short-term membrane fouling or removing viruses [39]; the concept and application of biological treatment are introduced to drinking water production as the growing polluted source of water. In biological treatment, extracellular polymeric substances (EPS) has played a major role during fouling formation [40] and an optimal concentration of bio-carrier might effectively reduce irreversible membrane fouling [29]. Khan *et al.* had quantified and demonstrated the dynamic effects of HRT, organic carbon, and EPS produced by microorganisms in a hybrid PAC-MF MBR as a sustainable technology for treating river water [41]. In addition, Jeong *et al.* employed a submerged membrane adsorption bioreactor (SMABR) as a pretreatment in seawater desalination for biofouling control and found that the SMABR was a sustainable biological pretreatment to reverse osmosis (RO) with only a small amount of PAC requirement [42]; Integrated pretreatments are designed and implemented in different types of industries and treating diverse water sources to utilize the advantages of each pretreatment to theoretically, or practically, enhance the membrane performance and control membrane fouling, to supplement each other’s disadvantages [4], such as ultrafiltration (UF) followed by reverse osmosis (RO) [43], UF followed by nanofiltration (NF) [44], integrated MBR-UF-RO system [45], TiO_2_ photocatalysis followed by MF, *etc.*, which could effectively reduce membrane fouling and enhance the permeate flux of the system. Effect of KMnO_4_ oxidation and FeCl_3_ coagulation on UF membrane fouling behavior was investigated by Tian * et al.*, and they found that in combination with low dosage FeCl_3_ coagulation, KMnO_4_ can further reduce total membrane fouling [46]. 

### 3.5. Brief Summary

Although each pretreatment measure has been used in potable water treatment and show certain effects, the problems of membrane fouling and flux decline in long-term operation are still limitations for large-scale application of membrane technology. (1) Coagulation pretreatment could remarkably improve filtration flux, reduce reversible fouling, lower the contents of colloids and NOM, while the dosage should be insured by tests and no obvious removal of small molecules occurring; (2) The adsorption pretreatment could improve filtration flux, notably remove small molecules, while it might also aggravate membrane fouling, is difficult to be eliminated and has a poor removal effect for macromolecular organics. Large studies might demonstrate that PAC is still active in the water treatment industry as an adsorbing material for the moment; (3) The oxidation pretreatment could improve filtration flux, lower the concentration of organic pollutants, reduce the possibility of biological contamination, while it might form the byproducts (such as halide acetate and trihalomethanes), and oxidize the membrane. The combination of membrane technology and pretreatment technologies are immature and only occur in several applications. Therefore, research on new technologies and methods to control membrane fouling, realize real-time monitoring, and optimize operation parameters will be the main research direction for a period of time. Moreover, the operation cost in the membrane separation technology is a key factor restricting its application, which should be given full consideration regarding the operation cost brought by medical and power consumption in pretreatment. When choosing the final design, the research should take the economized costs of prolonged membrane life span and reduced membrane cleaning times by pretreatment into consideration to achieve optimization of operation costs of the whole process, as multiple novel hybrid processes have emerged for water purification in raw water, municipal wastewater, recycled water, and sea-water treatment.

## 4. Hydrophilic Modification of Membrane

The most commonly used membrane material is an organic polymeric membrane, which contains Engineering polymer materials such as cellulose, polysulfone, polyacrylonitrile (PAN), polyvinylidene fluoride (PVDF), polyether ketone (PEK), polyimide(PI), *etc.* These materials have a chemical property of hydrophobicity, thus, membranes made from these materials are also strongly hydrophobic. In a practical application, the surface of hydrophobic membrane has no hydrogen-bond interaction with water; hydrophobic solutes approaching membrane surface have a spontaneous process of entropy increase, so they are easy to be absorbed to, and deposited on, the membrane surface, causing blockade of membrane holes. This can lead to severe membrane fouling, decrease membrane performance, and cut down on membrane life spans. Meanwhile, a single membrane material hardly possesses all the desirable properties including film-forming property, thermostability, chemical durability, acid-base resistance property, microbial erosion resistance, oxidative resistance, good mechanical strength, *etc.*, at the same time. As a result, the modification of membrane material or membrane surface is the common used way to improve its performance and to satisfy different requirements. The key step of membrane modification is introducing hydrophobic groups to the membrane surface. Various kinds of measures are applied to improve the hydrophilicity of membrane and anti-pollution capacity, increase membrane flux, and extend membrane life span. There are two categories of membrane modification: one is the modification of the membrane matrix, including blending and copolymerizing; the other is modification of the membrane surface, which introduces polar groups or grafting hydrophilic monomers to the membrane surface. The physical and chemical modifications on the membrane surface are also an effective way to improve membrane hydrophily and alleviate membrane fouling. The following parts summarized several kinds of membrane modification methods, including plasma modification, radiation grafting modification, surface coating modification, and material blending modification. A brief summary of three membranes materials upon the long-term performance, used for the manufacturing of commercial membranes, is given in Table 2.

**Table 2:** The morphology, hydrophobicity of three commercial membranes on long-term performance.

Commercial membranes	Hydrophobicity/hydrophilicity	Roughness	Flux decline
Polysulphone (PSf)	Hydrophobicity	More rough	Largest
Polyethersulphone (PES)	Hydrophobicity	Smoother	Less large
Regenerated cellulose (RC)	Hydrophilicity	Similar roughness to PSf	Smaller

### 4.1. Plasma Modification

The plasma grafting modification consists of pulsed plasma, consecutive plasma, and microwave plasma, and so on. During the modification, a membrane surface will be exposed to plasma to make free radicals and graft hydrophilic monomers, thus successfully modifying the membrane. Wang *et al.* modified polyvinylidene fluoride hollow fiber ultrafiltration membranes with low-temperature plasma, and grafted acrylic acid and acrylamide monomer to alleviate membrane fouling [47]. The low-temperature plasma modification method could graft acrylic acid and acrylamide monomer to the membrane surface effectively. After the modification, polar groups were introduced to the membrane surface. The modified membrane had a higher zeta potential than the original membrane. Kim *et al.* modified polyvinylidene fluoride membrane with low-temperature plasma to improve membrane hydrophily and make PA-PVDF flat composite membranes, and results showed that after being modified by plasma, the contact angle of polyvinylidene fluoride membrane decreased largely; the generated hydrophilic surface could be a supporting layer of TFC membrane and the composite membrane had a larger pure water flux [48]. Yang *et al.* did research on the low-temperature plasma modification of the flat ultrafiltration membrane of polyacrylonitrile (PAN) and results showed that the modified PAN had a lower water flux and a higher reject rate, and the modification degree could be controlled by changing conditions of low-temperature plasma [49]. Plasma treatment is a simple and convenient modification of membranes. However, the movement of polymer chains can lead polar groups transfer into polymer noumenon as time extends and temperature increases, which would result in the rebound of surface contact angle and affect the hydrophilic modification effect. The purity and ratio of gases should be strictly controlled when choosing plasma to trigger gas, so as to graft simplex reaction groups on the surface. After being modified with low-temperature plasma, membrane surfaces will be etched and the roughness will increase, which makes water easily spread out on the surface and this is one reason for a higher hydrophily of modified membranes. 

### 4.2. Radiation Grafting Modification

Radiation grafting modification is an important way to modify membrane surfaces. With γ-rays or electron beams, the polymer was exposed to high-energy radiation and then produced the active species. Active species triggered the monomer to process grafting polymerization. Groups or polymer chains with a certain characterization would be grafted to macromolecular chains of the membrane, thus the membrane can possess certain properties. Shim *et al.* grafted the hydrophilic methacrylate-2-hydroxyethyl acrylate monomer to a polypropylene ultrafiltration membrane surface with γ-rays, and then treated the modified membrane with bovine serum albumin solution [50]. They found that the newly-generated membrane had a higher flux, anti-fouling, and hydrophily. Higuchi *et al.* found that grafted Polyvinylpyrrolidone to Polysulfone with an immobilized amount of vinylpyrrolidone were the most hydrophilic membranes among the polysulfone and surface-modified polysulfone membranes [51].

### 4.3. Surface Coating Modification

By the way of coating a layer of hydrophilic substances, the surface coating modification can help to improve the hydrophily and anti-fouling capacity of membrane. Higuchi *et al.* coated a layer of hydrophilic Pluronict TM, which is a PEO-PPO-PRO Block copolymer, and found that the coated PSF membrane had a smaller adsorption of plasma proteins and platelets, which indicated that the new membrane with Pluronict TM coating had a higher anti-fouling capacity [52]. Revanur *et al.* coated amphiphilic polymer of polycyclooctene–polyethylene oxide (PEO) on the PVDF ultrafiltration membrane and found that when the coated membrane was processed with a light-induced cross-linking substance, its anti-peel capacity increased and the modified membrane had an improved anti-fouling capacity [53]. Hyun *et al.* coated the Polysulfone membrane with double amphiphilic comb polymer [54]. Under five circulations of screening–washing by microorganisms, alginic acid, and bovine serum albumin, results showed that the modified membrane had a better flux recovery than the original one, which indicated that coating with comb polymers can effectively reduce the membrane fouling made by biological macromolecules. Klibanov and Lewis *et al.* obtained nonleaching, permanent, sterile-surface materials that have been developed in which one end of a long-chained hydrophobic polycation, containing antimicrobial monomers, is attached covalently to the surface of a material, for example, cotton or plastic [55,56]. Ulbricht *et al.* obtained a polymer with an optimized block copolymer additive which exhibited ascendant bactericidal properties and surface properties [57]. Therien-Aubin *et al.* synthesized functionalized polyaramide membranes containing an atom transfer radical polymerization initiator as a versatile approach to easily modify the surface properties of the polyaramide, such as reducing the adhesion between the membrane and foulant [58]. In addition, Sagle *et al.* synthesized three series of copolymer hydrogel networks and found that such materials might improve the fouling resistance of membranes towards oily wastewater [59].

### 4.4. Blending Modification

The blending of high polymer materials means that two or more kinds of high polymer materials blend to generate a new kind of material, which has a comprehensive characterization of original materials as well as new outstanding properties that can overcome their respective defects. The membrane modified by high polymer blending has the following three advancements: a better hydrophily of membrane and the film-forming properties of the polymer, an improved anti-fouling capacity, and the increased physical-chemistry stability. Xin *et al.* blended PVC and IB-co-MH to create an alloy ultrafiltration membrane [60]. With the increase of IB-co-MH content, the contact angle of pure water decreased and the hydrophily rose. The addition of polymers increased the filtration flux as well as the removal rate, and the addition of hydrophilic substances mainly improved membrane’s permeation. Cherdon *et al.* blended HMA (The wholly aromatic polyamide) with hydrophilic polyvinylpyrrolidone to create a new membrane that has an enhanced hydrophily and anti-fouling capacity [61]. Recent work indicated that regenerated cellulose (RC) had a similar roughness to Polysulphone (PSf) but was much more hydrophilic [16]. In addition, the materials of PVDF blending with TiO_2_ nanoparticles has been widely explored, such as improving the thermal stability, hydrophilicity, antifouling, antibacterial, mechanical strength, and photocatalytic performance [62,63,64,65]. Wei *et al.* developed a new PVDF-TiO_2_ nanowire hybrid ultrafiltration membrane which can avoid some of the drawbacks of PVDF-TiO_2_ nanoparticle hybrid membrane [66]. A table was exhibited to summarize the state-of-the-art polymeric materials used for the manufacturing of commercial membranes by Ulbricht [67], and is given in Table 3. 

### 4.5. Brief Summary

All kinds of modifications of membranes can help to improve surface polarity, reduce contact angle, and increase surface energy. Various modification techniques have been developed, including the use of additives, chemical treatments, grafting components, and coatings. Each of these methods has its merits and demerits.

(1) The plasma modification is clean, effective, and pollution-free, but this kind of modification needs vacuum equipment. As such, it is unsuited for large-scale operation.
(2) The high-energy radiation has strength on its high use ratio of energy and its security. However, it is too powerful to control the reaction on the surface, which easily affects its original property.
(3) Coating hydrophilic substances on the membrane surface can further strengthen the modification effect and improve the membrane flux. Despite this advantage, the coating layer is easily sloughed off. As a result, the flux of pure water will firstly increase greatly and then decline gradually.

With the widespread use of ultrafiltration technology, high polymer substances will be the primary membrane materials used, and the research on its property should be focused. More kinds of modification methods will emerge when new materials are developed.

**Table 3:** Polymers as materials for industrially established separation membranes (cited from Ulbricht [67]).

Polymer	Morphology			Membrane process
	Barrier type	Cross-section	Barrier thickness (µm)	
Cellulose acetates	Nonporous	Anisotropic	~0.1	GS, RO
	Mesoporous	Anisotropic	~0.1	UF
	Macroporous	Isotropic	50–300	MF
Polyacrylonitrile	Mesoporous	Anisotropic	~0.1	UF
Polyetherimides	Mesoporous	Anisotropic	~0.1	UF
Polyethersulfones	Mesoporous	Anisotropic	~0.1	UF
	Macroporous	Isotropic	50–300	MF
Polyethylene terephthalate	Macroporous	Isotropic track-etched	6–35	MF
Polyphenylene oxide	Nonporous	Anisotropic	~0.1	GS
Poly(styrene-*co*-divinylbenzene), sulfonated or aminated	Nonporous	Isotropic	100–500	ED
Polytetrafluoroethylene	Macroporous	Isotropic	50–500	MF
	Nonporous	Isotropic	~0.1	GS
Polyamide, aliphatic	Macroporous	Isotropic	100–500	MF
Polyamide, aromatic	Mesoporous	Anisotropic	~0.1	UF
Polyamide, aromatic, *in situ* synthesized	Nonporous	Anisotropic/composite	~0.05	RO, NF
Polycarbonates, aromatic	Nonporous	Anisotropic	~0.1	GS
	Macroporous	Isotropic track-etched	6–35	MF
Polyether, aliphatic crosslinked, *in situ* synthesized	Nonporous	Anisotropic/composite	~0.05	RO, NF
Polyethylene	Macroporous	Isotropic	50–500	MF
Polyimides	Nonporous	Anisotropic	~0.1	GS, NF
Polypropylene	Macroporous	Isotropic	50–500	MF
Polysiloxanes	Nonporous	Anisotropic/composite	~0.1 < 1–10	GS PV, NF (organophilic)
Polysulfones	Nonporous	Anisotropic	~0.1	GS
	Mesoporous	Anisotropic	~0.1	UF
Polyvinyl alcohol, crosslinked	Nonporous	Anisotropic/composite	＜1-10	PV (hydrophilic)
Polyvinylidenefluoride	Mesoporous	Anisotropic	~0.1	UF
	Macroporous	Isotropic	50–300	MF

## 5. Perspectives of Further Research

Membrane separation technology has been widely used in the chemical industry, electronics, the light industry, textiles, and other industries. To further promote membrane application in the field of water treatment, the following aspects are proposed for further investigation.

(1) Mechanisms of impacts of coagulation, PAC adsorption, and ozone oxidation on membrane fouling and membrane filtration flux should be further studied to provide fundamental information and theoretical guidance for the understanding of membrane fouling mechanisms and the controlling of membrane fouling.
(2) Developing a new pretreatment technology can improve effluent quality and control membrane fouling economically and environmentally.
(3) Seek for the best feed conditions and membrane operation states to optimize operation effects.
(4) Accelerate the development of membrane modules with high filtration flux and low membrane blocking.
(5) Continue to develop functional polymer membrane materials. According to the knowledge of membrane separation mechanisms, the synthesis of various functional polymer molecules to produce homogenous membrane should be further carried out and the relationship between molecular structure and separation quality should also be studied quantitatively.
